# Seroprevalence of *Ehrlichia canis*, *Ehrlichia chaffeensis *and *Ehrlichia ewingii *in dogs in North America

**DOI:** 10.1186/1756-3305-5-29

**Published:** 2012-02-08

**Authors:** Melissa J Beall, A Rick Alleman, Ed B Breitschwerdt, Leah A Cohn, C Guillermo Couto, Michael W Dryden, Lynn C Guptill, Cristina Iazbik, Stephen A Kania, Patty Lathan, Susan E Little, Alma Roy, Katherine A Sayler, Brett A Stillman, Elizabeth G Welles, Wendy Wolfson, Michael J Yabsley

**Affiliations:** 1 IDEXX Laboratories, Inc., Westbrook, ME, USA; 2 University of Florida, College of Veterinary Medicine, Gainesville, FL, USA; 3 North Carolina State University, College of Veterinary Medicine, Raleigh, NC, USA; 4 University of Missouri, College of Veterinary Medicine, Columbia, MO, USA; 5 The Ohio State University, College of Veterinary Medicine, Columbus, OH, USA; 6 Kansas State University, College of Veterinary Medicine, Manhattan, KS, USA; 7 Purdue University, College of Veterinary Medicine, West Lafayette, IN, USA; 8 University of Tennessee, College of Veterinary Medicine, Knoxville, TN, USA; 9 Mississippi State University, College of Veterinary Medicine, Mississippi State, MS, USA; 10 Oklahoma State University, College of Veterinary Medicine, Stillwater, OK, USA; 11 Louisiana State University, College of Veterinary Medicine, Baton Rouge, LA, USA; 12 Auburn University, College of Veterinary Medicine, Auburn, AL, USA; 13 University of Georgia, College of Veterinary Medicine, Athens, GA, USA

**Keywords:** *Ehrlichia*, *E. canis*, *E. chaffeensis*, *E. ewingii*, dog, tick, prevalence

## Abstract

**Background:**

This study evaluated the exposure of dogs to three different *Ehrlichia *spp. in the south and central regions of the United States where vector-borne disease prevalence has been previously difficult to ascertain, particularly beyond the metropolitan areas.

**Methods:**

Dog blood samples (n = 8,662) were submitted from 14 veterinary colleges, 6 private veterinary practices and 4 diagnostic laboratories across this region. Samples were tested for *E. canis*, *E. chaffeensis *and *E. ewingii *specific antibodies using peptide microtiter ELISAs.

**Results:**

Overall, *E. canis*, *E. chaffeensis *and *E. ewingii *seroprevalence was 0.8%, 2.8%, and 5.1%, respectively. The highest *E. canis *seroprevalence (2.3%) was found in a region encompassing Arkansas, Louisiana, Oklahoma, Tennessee and Texas. *E. chaffeensis *seroreactivity was 6.6% in the central region (Arkansas, Kansas, Missouri, and Oklahoma) and 4.6% in the southeast region (Georgia, Maryland, North Carolina, South Carolina, Tennessee and Virginia). Seroreactivity to *E. ewingii *was also highest in the central region (14.6%) followed by the southeast region (5.9%). The geospatial pattern derived from *E. chaffeensis *and *E. ewingii *seropositive samples was similar to previous reports based on *E. chaffeensis *seroreactivity in white-tailed deer and the distribution of human monocytic ehrlichiosis (HME) cases reported by the CDC.

**Conclusions:**

The results of this study provide the first large scale regional documentation of exposure to *E. canis*, *E. chaffeensis *and *E. ewingii *in pet dogs, highlighting regional differences in seroprevalence and providing the basis for heightened awareness of these emerging vector-borne pathogens by veterinarians and public health agencies.

## Background

Dogs are susceptible to infection with multiple *Ehrlichia *spp., including *E. chaffeensis *and *E. ewingii*, which are predominantly transmitted by *Amblyomma americanum *(lone star tick), and to *E. canis*, whose primary vector is considered to be *Rhipicephalus sanguineus *(brown dog tick) [1-4]. *Amblyomma americanum *is commonly found on dogs and people in the southeastern and southcentral United States; indeed, human monocytic ehrlichiosis caused by *E. chaffeensis *is the most frequently diagnosed tick-borne disease in the southern U.S. [5,6]. The number of human monocytic ehrlichiosis cases reported annually has also risen steadily from 200 patients in the year 2000 to over 900 patients in 2009 [7,8]. Infections with *E. ewingii *are also well documented in both dogs and humans in the southeastern and southcentral U.S. [4,9-12]. The geographic range of *A. americanum *has expanded dramatically in recent decades to include many areas of the Midwest and Northeast, resulting in a concomitant increase in the regions at risk for autochthonous transmission of *E. chaffeensis *and *E. ewingii *to dogs and people [6,13]. Investigative field surveys have implicated other tick species, including *Dermacentor variabilis *(American dog tick) and *R. sanguineus*, which have been implicated as secondary vectors of *E. chaffeensis *and/or *E. ewingii *[14-16], but the relative importance of these ticks in maintaining a sustained cycle of infection in nature remains to be determined.

*Rhipicephalus sanguineus *ticks, the vectors for *E. canis*, are also common in the warmer climates of the southern U.S., due in part to their tropical or Mediterranean origin and general intolerance of cold temperatures [17,18]. Brown dog ticks are thought to have been introduced to the Americas, most likely on tick infested dogs from Europe. Because this tick can survive inside dwellings wherever dogs are present, *R. sanguineus *is now considered to be endemic throughout the U.S. with higher prevalence in particular geographic regions [19-21]. Brown dog ticks are known or strongly suspected to transmit a number of other pathogens to dogs in North America, including *Bartonella vinsonii *subsp. *berkhoffii*, *Rickettsia rickettsii*, *Babesia canis*, *Hepatozoon canis*, and *Anaplasma platys *[17,22].

Diagnosis of an ehrlichial infection can be performed using visual, serologic or molecular methods. *Erhlichia *spp. replicate inside a membrane bound vacuole (i.e., morula) that can sometimes be observed by light microscopic examination of stained blood smears inside either monocytes (*E. canis *and *E. chaffeensis) *or granulocytes (*E. ewingii*). Detection of antibodies can be performed by immunofluorescent assay (IFA) or enzyme linked immunosorbant assays (ELISA) but cross-reactivity between antibodies to *Ehrlichia *species is possible [23]. Polymerase chain reaction (PCR) is the most common molecular method used to diagnose an *Ehrlichia *spp. infection, particularly in dogs with acute illness where the onset of clinical signs may precede a measurable antibody response.

Serologic surveys of dogs for exposure to tick-borne pathogens have been used to identify areas where both people and dogs are at risk of acquiring infection [24-28]. However, prospective surveys involving dogs have often been limited to a small geographic region, such as a portion of a single state [29-31]. Reports of large scale retrospective analyses may be skewed to areas with higher human population density and thus higher pet dog density. However, these regions may not represent regions with the highest tick exposure. For example, in a recent publication describing exposure to common tick-borne pathogens in nearly one million dogs from the U.S. [25], only 6.4% of samples came from the states of Kansas, Oklahoma, Missouri, Arkansas, Louisiana, and Mississippi, where very dense populations of *A. americanum *ticks are found. In addition, the majority of results reported were from urban areas within the states. To combat the bias inherent in such surveys and to better document the prevalence of exposure to *Ehrlichia *spp. in the south and central U.S., samples collected from dogs presenting primarily to regional veterinary colleges were tested for *E. canis*, *E. chaffeensis *and *E. ewingii *specific antibodies. A better understanding of the distribution of exposure to these three ehrlichial agents in dogs will provide insight into areas where people and dogs are at greatest risk of infection.

## Methods

### Canine Serum Samples

A total of 8,662 canine serum samples were collected from 14 veterinary colleges, 4 commercial diagnostic laboratory locations and 6 private veterinary practices. Participating veterinary colleges included Auburn University, University of Florida, University of Georgia, University of Illinois, Kansas State University, Louisiana State University (LSU), Mississippi State University, University of Missouri, North Carolina State University (NCSU), The Ohio State University, Oklahoma State University (OKSU), Purdue University, University of Tennessee, and Texas A&M University (TAMU). Participating commercial diagnostic laboratories were located in Dallas, TX, Baltimore, MD, Totowa, NJ, and North Grafton, MA. The private practices were located in Arkansas, Missouri, North Carolina and Tennessee. The majority of samples collected from veterinary colleges and from all diagnostic laboratories consisted of serum that remained after performance of requested diagnostic tests unrelated to this study. These samples were chosen randomly, without regard to clinical signs or suspected diagnosis, from among all remaining serum samples from dogs ≥ 6 months of age. Serum samples collected from dogs involved in shelter medicine programs represented all samples from Texas A&M and 183/307 serum samples from Louisiana State University. Samples submitted from NCSU included an equal number (250 each) of randomly chosen samples and samples previously submitted to the NCSU Vector-Borne Disease Diagnostic Laboratory (VBDDL) for serological testing. More than half of the serum samples collected from OKSU (360/503) were originally submitted for brucellosis testing. For samples submitted from private veterinary practices, a small additional volume of blood (approximately 2 ml) was collected with informed owner consent specifically for the purposes of this study at the time of blood collection related to routine veterinary care; sampled dogs were chosen from the general population and not selected based on particular clinical signs. Although most samples were collected between January 2009 and October 2010, 250 samples from the NCSU-VBDDL were collected during the first 6 months of 2006.

### Data collection

Limited patient information was available for the samples. However, the date of sample collection, the dog's actual or approximate age, and the address zip code of the dog's owner, veterinary hospital or shelter was recorded. Neither breed nor gender of dogs was recorded.

### Serology

All serum samples were tested by 3 microtiter plate ELISAs, each one using a species-specific peptide for the detection of antibodies reactive to *E. canis*, *E. chaffeensis *and *E. ewingii*. Synthetic peptides were derived from the *E. ewingii *p28 protein (EESP), the *E. canis *p16 protein, and the *E. chaffeensis *variable-length PCR target (VLPT) protein [32,33]. Testing was performed as previously described with minor modifications [32]. Samples were initially screened on each of the 3 microtiter plate ELISAs using an indirect assay protocol and confirmed using the same peptide ELISA but with a peptide-labeled direct assay. Briefly, in the indirect assay, diluted samples (1:100) were incubated in each of the 3 species-specific ELISA peptide-coated wells for 30 minutes, the wells were washed (300 μL/well with PBS plus detergent), and horseradish peroxidase-labeled anti-canine antibody (1:1000 dilution in a diluent containing non-specific protein and detergent) was added to each well and incubated for 30 minutes. Plates were washed as above, 3,3',5,5' teteramethylbenzidine (TMB) substrate solution (50 μL/well) was added, and optical density was determined at 650 nm; reactive samples were denoted by any absorbance value > 2 times the negative control value. Reactive samples were confirmed in duplicate by adding 50 ul of the serum sample and 100 μL of a species-specific peptide horseradish peroxidase conjugate (0.5 to 2.0 μg/mL in diluent) to the microtiter-plate ELISA coated with the species-specific peptide. This was allowed to incubate for 60 min, microtiter wells were then washed 6 times, incubated with TMB substrate (100 μL/well) and read at 650 nm as described above.

Results for *E. canis *immunofluorescence assays (IFA) were available for 249 samples from the VBDDL at NCSU. Testing was performed following previously described standard procedures of the VBDDL service [34]. *E. canis *antigens (NCSU Jake strain) were grown *in vitro *in DH82 cells by the VBDDL. Seropositive samples were defined as having endpoint titers ≥64 using a dilution scale of 1:16 - 1:8192.

### Analysis

Seroprevalence for each pathogen was determined by county and state using zip code data of the patient, shelter or veterinary practice submitting the sample. At least 50 samples were required from a state in order to calculate seroprevalence as depicted in the maps. Seroprevalence results by county were calculated if at least 5 samples were obtained from a county. Geographic regions for comparison of seroprevalence were determined according to the expected tick population pressure. For seroprevalence of *E. canis*, which is transmitted primarily by *R. sanguineus*, the region with the greatest expected tick pressure was defined to include Alabama, Arkansas, Florida, Georgia, Louisiana, Mississippi, North Carolina, Oklahoma, South Carolina, Tennessee and Texas and this region was compared to all other states. For seroprevalence of *E. ewingii *and *E. chaffeensis*, which are transmitted primarily by *A. americanum*, the regions expected to have the greatest *A. americanum *pressure were defined as central (Arkansas, Kansas, Missouri, and Oklahoma), or southeastern (Georgia, Maryland, North Carolina, South Carolina, Tennessee and Virginia), and these two regions were compared to all other states. Comparison of seroprevalence between two geographic regions, as well as comparisons between groups of dogs, was made using the Chi-square test (*P *< 0.05 considered significant) while a one-way ANOVA with significance assigned at *P *< 0.05 was used for comparisons of 3 or more regions. Rates of HME per million in the population were calculated based upon the reported cases per state for the years 2008-2009 divided by the U.S. census data per state for the same years. Comparison of HME rates to *Ehrlichia *spp. seroprevalence was performed using a linear regression for those states with at least 50 canine samples and the coefficient of determination (R^2 ^) was calculated. Analyses were performed using GraphPad Prism v.5 (GraphPad Software, La Jolla, CA).

## Results

The 8,662 canine serum samples originated from dogs in 41 states, with more than 600 samples from each of the states of Texas, Florida, Missouri and Georgia (Table 1). Many universities enrolled in the study collected at least 300 samples with the most being submitted by the University of Florida (Table 2). The average age of the dogs sampled was 6.6 years (range 0.5 - 23 years). The average age of dogs residing in shelters was younger (3.4 years) than the other dogs sampled (7.0 years) as was the age of dogs tested for brucellosis (3.1 years).

**Table 1:** Distribution of canine samples by state and *Ehrlichia *spp. seroprevalence relative to reported cases of HME.

State	Total Number Samples	Total *Ehrlichia *(%)	*E. canis* (%)	*E. ewingii *(%)	*E. chaffeensis *(%)	HME cases/M*
**Alaska**	**2**	**0**	**0**	**0**	**0**	**N**
Alabama	337	15 (4.5)	1 (0.3)	12 (3.6)	5 (1.5)	1.8
**Arkansas**	**84**	**37 (44.0)**	**3 (3.6)**	**31 (36.9)**	**18 (21.4)**	**21.7**
Arizona	1	0	0	0	0	0
**California**	**5**	**0**	**0**	**0**	**0**	**0.04**
Colorado	5	0	0	0	0	N
**Connecticut**	**97**	**0**	**0**	**0**	**0**	**0.3**
Delaware	27	0	0	0	0	23.3
**Florida**	**733**	**27 (3.7)**	**4 (0.5)**	**19 (2.6)**	**9 (1.2)**	**0.6**
Georgia	662	51 (7.7)	1 (0.2)	36 (5.4)	23 (3.5)	1.9
**Hawaii**	**2**	**0**	**0**	**0**	**0**	**N**
Iowa	14	0	0	0	0	N
**Illinois**	**489**	**17 (3.5)**	**0**	**8 (1.6)**	**9 (1.8)**	**2.4**
Indiana	553	4 (0.7)	0	4 (0.7)	3 (0.5)	0.3
**Kansas**	**457**	**35 (7.7)**	**4 (0.9)**	**31 (6.8)**	**5 (1.1)**	**1.1**
Kentucky	16	3 (18.8)	1 (6.3)	3 (18.8)	0	2.9
**Louisiana**	**274**	**6 (2.2)**	**4 (1.5)**	**2 (0.7)**	**0**	**0**
Massachusetts	241	2 (0.8)	0	2 (0.8)	2 (0.8)	2.3
**Maryland**	**254**	**16 (6.3)**	**2 (0.8)**	**9 (3.5)**	**9 (3.5)**	**8.3**
Maine	13	0	0	0	0	0.8
**Michigan**	**9**	**0**	**0**	**0**	**0**	**0.5**
Minnesota	7	0	0	0	0	2.1
**Missouri**	**663**	**190 (29)**	**5 (0.8)**	**151 (22.8)**	**85 (12.8)**	**28.4**
Mississippi	151	9 (6.0)	0	9 (6.0)	2 (1.3)	1.0
**Montana**	**1**	**0**	**0**	**0**	**0**	**N**
North Carolina	403	41 (10.2)	3 (0.7)	21 (5.2)	23 (5.7)	4.7
**Nebraska**	**62**	**1**	**1 (1.6)**	**0**	**0**	**1.4**
New Hampshire	28	0	0	0	0	4.2
**New Jersey**	**257**	**10 (3.9)**	**1 (0.4)**	**7 (2.7)**	**5 (1.9)**	**9.0**
New York	188	3 (1.6)	0	3 (1.6)	1 (0.5)	3.7
**Ohio**	**428**	**5 (1.2)**	**4 (0.9)**	**1 (0.2)**	**0**	**1.0**
Oklahoma	514	47 (9.1)	9 (1.8)	37 (7.2)	5 (1.0)	33.2
**Pennsylvania**	**96**	**1 (1.0)**	**0**	**1 (1.0)**	**0**	**0.7**
Rhode Island	24	0	0	0	0	9.0
**South Carolina**	**34**	**7 (20.6)**	**3 (8.8)**	**2 (5.9)**	**3 (8.8)**	**0.3**
South Dakota	2	0	0	0	0	0
**Tennessee**	**181**	**18 (9.9)**	**5 (2.8)**	**14 (7.7)**	**5 (2.8)**	**10.9**
Texas	893	23 (2.6)	18 (2.0)	5 (0.6)	1 (0.1)	0.7
**Virginia**	**385**	**48 (12.5)**	**2 (0.5)**	**31 (8.1)**	**25 (6.5)**	**8.4**
Wisconsin	9	0	0	0	0	4.0
**West Virginia**	**30**	**1**	**0**	**0**	**1 (3.3)**	**0.3**
Unknown	31	1	0	0	1	
**Total**	**8662**	**618 (7.1)**	**71 (0.8)**	**439 (5.1)**	**240 (2.8)**	

*Average incidence of HME cases per million as reported for 2008 and 2009 by passive surveillance; MMWR June 25, 2010 (2008) and May 13, 2011 (2009) (N - not reported)

**Table 2:** Number of canine samples submitted by institution and average patient age.

Location	Number of samples tested	Average patient age (yrs)
Auburn University	538	7.2
University of Florida	645	8.3
University of Georgia	514	8.0
University of Illinois	425	8.0
Kansas State University	521	7.4
Louisiana State University	307	3.7
Mississippi State University	182	7.0
University of Missouri	614	8.0
North Carolina State University	500	7.1
Oklahoma State University	503	3.1
The Ohio State University	501	NA
Purdue University	609	8.2
Texas A&M University	381	3.1
University of Tennessee	108	6.2
Private Veterinary Clinics (6)	314	6.1
Commercial Laboratories (4)	2000	7.0

(NA = not available)

Of the 8,662 samples tested, 618 (7.1%) were determined to have antibodies to at least one of the three *Ehrlichia *species. Antibodies to *E. canis*, *E. chaffeensis *or *E. ewingii *were detected in 71 (0.8%), 240 (2.8%) and 439 (5.1%) samples, respectively (Table 1). In 132 samples, antibodies to more than one *Ehrlichia *species were detected. The majority of these co-exposed dogs had antibodies to both *E. ewingii *and *E. chaffeensis *(121/132; 92%). Seven dogs had antibodies to *E. ewingii *and *E. canis*, 3 dogs had antibodies to *E. canis *and *E. chaffeensis *and only one dog was found to have antibodies to all three *Ehrlichia *species. Dogs with antibodies to *E. chaffeensis *were significantly more likely to have antibodies to *E. ewingii *than were either dogs seronegative for *E. chaffeensis *(*P *< 0.0001) or dogs seropositive for *E. canis *(*P *= 0.0051).

**Figure 1:**
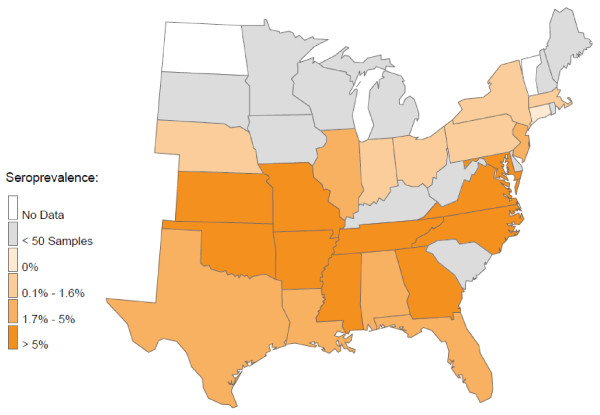
**Seroprevalence by state of all three *Ehrlichia *spp., *E. canis*, *E. chaffeensis *and *E. ewingii*, in dogs**.

**Figure 2:**
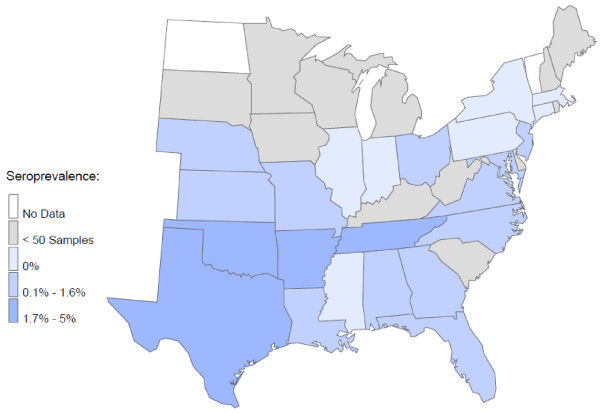
**Seroprevalence by state of *E. canis *in dogs**.

**Figure 3:**
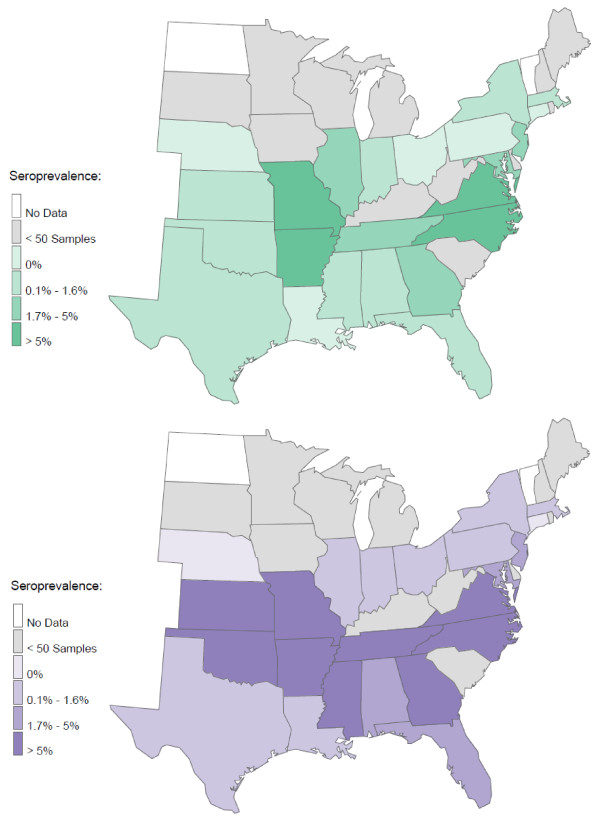
**Seroprevalence by state of *E. chaffeensis *(green) and *E. ewingii *(purple) in dogs**.

**Figure 4:**
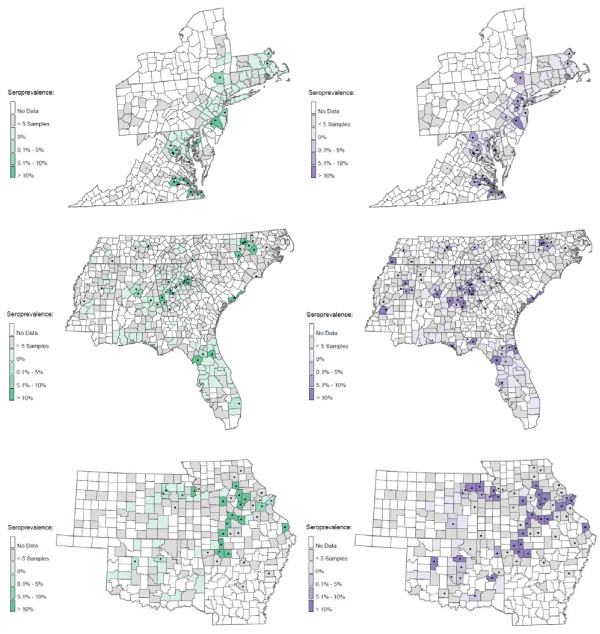
**Seroprevalence by county of *E. chaffeensis *(green) and *E. ewingii *(purple) in dogs for the mid-Atlantic, southeastern and central regions of the U.S**.. A black dot indicates that at least one seroreactive sample was identified in that county.

At least one seroreactive sample was identified from each of 25 states, and seroreactivity for at least 2 of the 3 *Ehrlichia *species was found in samples from 22 states (Figure 1). The region expected to have the greatest *R. sanguineus *tick pressure (Alabama, Arkansas, Florida, Georgia, Louisiana, Mississippi, North Carolina, Oklahoma, South Carolina, Tennessee and Texas) did not have significantly more *E. canis *seropositive samples (1.2%) compared to all other states combined (0.5%; *P *= 0.07) (Figure 2). However, the central portion (Arkansas, Louisiana, Oklahoma, Tennessee, Texas) of this region had significantly more samples seroreactive for *E. canis *than the eastern portion (Alabama, Florida, Georgia, Mississippi, North Carolina, South Carolina) of this region (2.0% vs. 0.5% respectively; *P*= 0.0121). Coincident with *A. americanum *distribution, seroreactivity to *E. chaffeensis *(Figures 3, 4) was 6.6% in the central region (Arkansas, Kansas, Missouri, and Oklahoma) and 4.6% in the southeast region (Georgia, Maryland, North Carolina, South Carolina, Tennessee and Virginia). Seroprevalence of *E. chaffeensis *in both of these *A. americanum *indigenous regions was significantly higher than the seroprevalence from all other states combined (0.7%; *P *< 0.0001). However, seroreactivity to *E. ewingii *(14.6%) was significantly higher in the central region compared with the seroprevalence in the southeastern region (5.9%) and when compared to all other states combined (1.4%; *P *< 0.0001) (Figures 3, 4).

**Figure 5:**
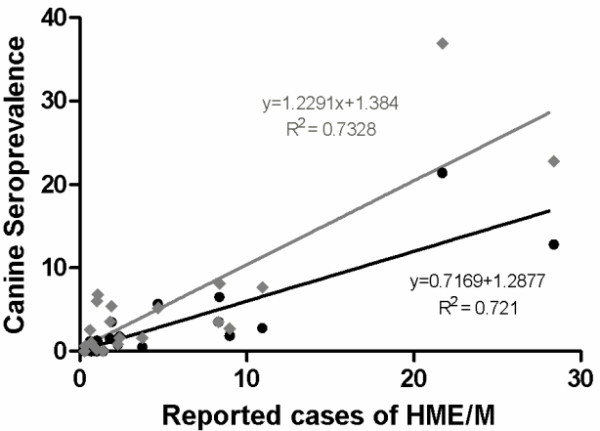
**Correlation between the average number of reported cases of HME/million people by state and seroprevalence for *E. chaffeensis *(grey) and *E. ewingii *(black) in dogs by state**. Analysis excluded HME rates and canine seroprevalence from those states where the veterinary institutions had a biased collection of samples from young dogs within the population.

To determine if the seroprevalence of *E. chaffeensis *and *E. ewingii *in dogs might correlate with the number of HME cases reported per 1,000,000 in the human population, a linear regression was performed on the data. If samples from all states were included, the coefficient of determination (R^2^) was only 0.47 and 0.37 for *E. ewingii *and *E. chaffeensis*, respectively. However, when the samples from dogs with significantly lower average age (those from LSU, OKSU, TAMU) were omitted from the analysis, the coefficient of determination increased to 0.72 and 0.73 for *E. ewingii *and *E. chaffeensis*, respectively (Figure 5). There was a significant correlation between HME cases reported per million people and seroprevalence of *E. ewingii *and *E. chaffeensis *in dogs (*P *< 0.0001) but not with seroprevalence of *E. canis *in dogs (*P *= 0.704).

*E. canis *IFA results were compared with that of the *Ehrlichia *species-specific ELISAs for 249 samples obtained from the NCSU-VBDDL. *E. canis *IFA identified 5 seropositive samples with titers of greater than or equal to 1:64. The *Ehrlichia *species ELISAs identified the same 5 samples as seropositive with 3 seroreactive for *E. canis*, one seroreactive for *E. chaffeensis*, and one seropositive for both *E. chaffeensis *and *E. ewingii*. The peptide ELISAs detected 20 additional *Ehrlichia *seroreactive samples within the group that had been negative by *E. canis *IFA. Of these 20, only 2 were seropositive for *E. canis *and the remainder had detectable antibodies to *E. chaffeensis*, *E. ewingii*, or both (Table 3). In this sample set, the *E. canis *IFA did not detect the *E. ewingii*-only seroreactive samples (n = 7) identified by the species-specific peptide ELISA.

**Table 3:** Comparison of serologic results using *E.canis *IFA and the three *Ehrlichia *species ELISAs

*Ehrlichia *species by ELISA:	*E. canis *IFA/*Ehrlichia *species ELISAs			
	
	Pos/Neg	Pos/Pos	Neg/Pos	Neg/Neg
*E. canis*	0	3	2	
*E. ewingii*	0	0	7	
*E. chaffeensis*	0	1	6	
*E. ewingii *+ *E. chaffeensis*	0	1	5	
**Total**	**0**	**5**	**20**	**224**

A total of 249 serum samples from the VBDDL at NCSU had previously been tested by *E. canis *IFA. Titers of 1:64 and greater were considered positive.

## Discussion

This study evaluated the exposure of dogs to three *Ehrlichia *species, with a particular focus on dogs from the south and central regions of the U.S., using peptide-based species-specific ELISA assays. Based on this study, dogs were most commonly exposed to *E. ewingii *with antibodies identified in 5.1% of all samples tested. Antibodies to *E. chaffeensis *were identified in 2.8% of dogs tested. This predominance of *E. ewingii *infection in dogs has been reported previously in studies from Missouri, Arkansas, and Oklahoma [4,10]; the present study confirms and extends that finding over a much wider geographic area. Antibodies to *E. ewingii *and *E. chaffeensis *were identified in dogs from 23 and 20 of the 41 states considered, respectively, suggesting a widespread distribution of these agents in the eastern U.S. This pattern parallels the distribution and intensity of *A. americanum *ticks in nature [35]. Further, it is consistent with data derived by less specific serologic assays using sera from deer and dogs [25,35,36].

The geographic distribution of *E. chaffeensis *and *E. ewingii *seroreactive dogs in the present study is also largely consistent with reports of HME or *E. ewingii *infections in people (see Table 1). However, the correlation with HME was not identified unless the institutions with a biased selection of young dogs in the population (shelter and kennel/breeder dogs) were excluded from the analysis suggesting that canine exposure may be influenced by the time spent in an endemic environment. A review of individual states revealed some instances where the *E. chaffeensis *and *E. ewingii *seroprevalence in dogs exceeded the reported number of HME cases in people. For instance, *E. chaffeensis *and *E. ewingii *were identified in approximately the same proportion of dogs tested from Kansas (1.1%; 6.8%) and Mississippi (1.3%; 6.0%) as from Tennessee (2.8%; 7.7%) but no cases of HME were reported from Kansas or Mississippi in 2008 whereas 64 HME cases were reported in people from Tennessee in that year [37]. Results from 2009 show an increase in the number of HME cases in Kansas and Mississippi, with each reporting 6 clinical cases, while 73 were reported from Tennessee [7]. Disease reporting often varies from year to year, and underreporting of HME is thought to commonly occur [5]. Alternatively, infection with other *Ehrlichia *spp. may be responsible for the high number of HME patients reported from regions with low canine seroprevalence as novel *Ehrlichia *spp. continue to be described [38-40].

Interestingly, 121 (1.4%) dogs in the present study had antibodies to both *E. ewingii *and *E. chaffeensis*, suggesting some degree of simultaneous or sequential infection with these two agents, both of which are transmitted primarily by *A. americanum *ticks. Co-infection in dogs with multiple *Ehrlichia *spp. has been reported from Missouri and North Carolina, based upon molecular methods [10,34]. Human co-infection with *E. chaffeensis *and *E. ewingii *has not been reported, but the findings from the present study, the shared vector tick, and the inability to distinguish infections with these two agents by less specific serologic techniques, such as IFA, suggests co-infection should be considered in people, particularly when more severe disease is evident. Experimental infection with multiple rickettsial agents in dogs has been shown to lead to more severe disease than infection with a single pathogen [41].

*E. canis *is a well recognized tick-borne pathogen of dogs known to cause monocytic ehrlichiosis. In this study exposure to *E. canis *was highest in the region of Texas, Louisiana, Arkansas and Oklahoma, which is consistent with the density of the primary tick vector, *R. sanguineus*. However, the overall seroprevalence of *E. canis *was much lower compared to the seroprevalence of *E. chaffeensis *and *E. ewingii*, which may be consistent with the increase in *A. americanum *numbers and expanding geographic distribution of this tick species [5]. Additionally, previous serosurveys which have utilized methods like *E. canis *IFA or the SNAP^®^3Dx^® ^or SNAP^®^4Dx^® ^test kits to measure *E. canis *seroprevalence may have detected ehrlichial antibodies other than those specific for *E. canis *resulting in an overestimate of *E. canis *exposure [23,42]. This differs from the present study which utilized a peptide that is specific for *E. canis *and does not detect antibodies to *E. chaffeensis *or *E. ewingii *[32]. Finally, the advent of more efficacious and long-acting acaracides could be having an impact on *E. canis *exposure rates in dogs due to the resulting suppression of the natural maintenance cycle for *E. canis*. Both the greater host specificity of *R. sanguineus *ticks relative to *A. americanum *and the lack of wildlife reservoir for *E. canis *favor a suppression of the natural maintenance cycle for *E. canis *relative to *E. chaffeensis *and *E. ewingii*. No such suppression of *A. americanum *maintenance cycles in deer or other wildlife inhabiting natural environments would be expected to be ongoing currently.

In addition to the results of this study, results from previous experimental infection studies with *E. chaffeensis *and *E. ewingii *[32] as well as regional field studies [4] have demonstrated the sensitivity and specificity of these peptide reagents for antibody detection in infected canine samples. To further evaluate the performance, a limited number of samples were used to compare the results of the three *Ehrlichia *ELISAs with *E. canis *IFA as performed by the NCSU VBDDL. When compared to *E. canis *IFA, 20/249 samples (8%) were reactive on the species-specific ELISAs that did not demonstrate reactivity by IFA testing. Results of a previous study demonstrated that up to 8% of samples with reactivity to *Ehrlichia *species peptide reagents on SNAP 3Dx (p30/p30-1) may test negative (titers < 1:64) on *E. canis *IFA [23]. The identification of samples having antibodies to *E. chaffeensis *and/or *E. ewingii *by ELISA but not confirmed positive by *E. canis *IFA may reflect the variability of cross-reactive antibodies between these *Ehrlichia *species. Differences in assay format between ELISA and IFA, such as testing a less dilute serum sample and the use of highly concentrated immunodominant epitopes on the ELISA, may account for some of the observed differences. More studies are needed to determine the degree with which cross-reacting antibodies in dogs exposed to *E. chaffeensis *and *E. ewingii *will react with *E. canis *IFA, the primary *Ehrlichia *species used as antigen in IFA testing by veterinary diagnostic laboratories.

## Conclusions

In this study, we documented that dogs in the central and south central U.S. are more commonly exposed to *E. ewingii *than other ehrlichial species, and are more commonly exposed to *E. chaffeensis *than to *E. canis*. In the case of *E. chaffeensis *and *E. ewingii*, the lone star tick transmits these infectious agents to both dogs and people. The dog has been described as a sentinel for vector-borne infections like Lyme Disease and Rocky Mountain Spotted Fever [29,43-45]. The results of this study provide preliminary evidence that dogs can be tested using *Ehrlichia *species-specific peptides and serve as a regional or local sentinel to gauge the potential risk for human infection with these tick-transmitted pathogens.

## Competing interests

MJB, and BS are employees of IDEXX Laboratories, Inc. EBB, LAC, MAD, SEL, and MJY have received funding from IDEXX Laboratories, Inc. for education, research or consulting.

## Authors' contributions

MJB, SEL and LAC were primarily responsible for the first draft of the manuscript. All authors critically reviewed and approved the final manuscript.
